# Three-dimensional analysis of maxillary asymmetry in patients with mandibular prognathism

**DOI:** 10.1186/s40902-026-00506-y

**Published:** 2026-06-02

**Authors:** Chihiro Atarashi, Kazuaki Osawa, Daisuke Saito, Hideyoshi Nishiyama, Kei Tomihara, Isao Saito, Jun Nihara

**Affiliations:** 1 Division of Orthodontics, Faculty of Dentistry & Graduate School of Medical and Dental Sciences, Niigata University, Niigata, Japan; 2 Department of Oral and Maxillofacial surgery/Orthodontics, Yokohama City University Hospital, Yokohama, Japan; 3 Division of Reconstructive Surgery for Oral and Maxillofacial Region, Faculty of Dentistry & Graduate School of Medical and Dental Sciences, Niigata University, Niigata, Japan; 4 Division of Oral and Maxillofacial Radiology, Faculty of Dentistry & Graduate School of Medical and Dental Sciences, Niigata University, Niigata, Japan; 5 Division of Oral and Maxillofacial Surgery, Faculty of Dentistry & Graduate School of Medical and Dental Sciences, Niigata University, Niigata, Japan

**Keywords:** 3-dimensional analysis, Cluster analysis, Jaw deviation, Mandibular prognathism, Maxillary morphology

## Abstract

**Background:**

Jaw deviation is a common feature of skeletal mandibular prognathism and often extends to the maxilla, complicating diagnosis and treatment planning in surgical orthodontic treatment. Since occlusion and maxillary morphology strongly influence mandibular position and facial balance, accurate evaluation of maxillary asymmetry is essential. Three-dimensional computed tomography (3D-CT) is more reliable than conventional two-dimensional cephalometric analysis, which is limited by distortion and structural overlap. This study aimed to quantify maxillary deviation in yawing and rolling directions and to classify morphological patterns using cluster analysis of 3D-CT data.

**Results:**

One hundred patients with skeletal mandibular prognathism were analyzed using 3D-CT. Eleven anatomical landmarks were digitized, and twelve asymmetry indices were calculated. Cluster analysis of yawing deviation produced three groups: mild deviation (70 patients), moderate deviation (23 patients), and severe deviation (7 patients). Rolling deviation also yielded three groups: mild deviation (39 patients), moderate deviation (52 patients), and severe deviation (9 patients). Combined analysis classified all cases into nine subgroups. Of those, 31% showed nearly symmetrical morphology, while 69% demonstrated varying degrees of maxillary asymmetry. Characteristic patterns included rolling deviation at the greater palatine foramen, yawing deviation in the posterior alveolar process, and mandibular fossa deviation with mild rolling features. Less frequent subgroups reflected complex and severe morphologies.

**Conclusion:**

Approximately two-thirds of patients with mandibular prognathism exhibited maxillary asymmetry, predominantly in posterior alveolar and basal regions. Classification into nine subgroups clarified distinct deviation patterns, with four groups demonstrating clinically relevant characteristics. These results emphasize the critical role of maxillary morphology in jaw deviation and highlight the value of standardized 3D evaluation. Incorporating maxillary asymmetry patterns into treatment planning may enhance diagnostic accuracy, improve surgical orthodontic outcomes, and contribute to more predictable facial symmetry.

## Background

Jaw deviation is often observed along with significant anterior–posterior, vertical, and horizontal combined structural abnormalities in jaw deformities for which surgical orthodontic treatment is applied [1]. There are many variations in the aspect of jaw deviation, and the deviation may extend not only to the mandible, this is easily recognized, but also to the maxilla, because the maxillofacial skeleton and dentition in facial asymmetric cases is a conglomeration of three-dimensional structures with a high degree of three-dimensional complexity [2]. According to previous studies, a high frequency of jaw deformities with jaw deviation includes improvement of facial asymmetry as the patient's motivation for treatment [3], and therefore, the acquisition of facial symmetry is one of the most important treatment goals in surgical orthodontic treatment.

It is necessary to plan treatment after understanding of the aspect of jaw deviation in order to improve the facial asymmetry in jaw deformities. Regarding the origin of jaw deviation, some researchers suggest that occlusion and maxillary position also affect mandibular position [4]. In addition, it has been pointed out that in most patients with facial asymmetry, the mandible may gradually deviate to the upper cant side of the maxilla [5], so maxillary deviation is important for understanding of the aspect of jaw deviation.

It is difficult to accurately evaluate maxillary deviation by soft tissue because asymmetric bony tissue is often hidden by soft tissue [6]. Therefore, maxillary deviation has been evaluated by horizontal occlusal plane inclination in the frontal cephalogram analysis and the intersection of the left and right maxillary tuberosities and the zygomatic alveolar ridge (J-J') [7]. However, frontal cephalometric, a two-dimensional (2D) analysis method, can be a source of misdiagnosis. The frontal cephalogram is not reliable because many important parameters cannot be measured with a planar cephalogram. And facial asymmetry causes image magnification and distortion in most 2D cephalometric measurements, it is affected by the position of the head during radiography, and skeletal structures may overlap [8–10]. Therefore, three-dimensional analysis is essential to characterize jaw deviation, a three-dimensional morphological abnormality [8, 11, 12]. Three-dimensional computed tomography (3D-CT) images are used for three-dimensional analysis of skeletal structures. The measurements taken from 3D-CT images are close to those obtained from the dried skull and are sufficiently accurate [13, 14].

The setting of the midsagittal plane is quite important for the three-dimensional analysis of the jaw, especially jaw deviations. It is essential to establish anatomical reference points that are as unaffected as possible by jaw deviation for appropriate evaluation. Various studies about setting up a three-dimensional median sagittal reference plane have been conducted in the past [12, 15].

There have been attempts at three-dimensional evaluation of jaw deviation involving the maxilla [16–19], but few have focused on the maxilla, and it is difficult to evaluate the complex aspects of deviation, such as evaluation based on the teeth rather than the bone, and cases in which there is no mid-dentition deviation but jaw deviation occurs.

Therefore, if the aspect of deviation of the maxilla is quantified three-dimensionally and similar patterns are identified, it is expected leading to the standardization of the treatment system, which has been based on the aspect of deviation of each case, and to the provision of higher quality and homogeneous surgical orthodontic treatment.

Cluster analysis, which analyzes the characteristics of groups obtained by classifying cases into several groups and similarizing them on a statistical basis according to objective numerical criteria, is effective in understanding the aspect of deviation for jaw deviations with many variations in morphology [20]. On the other hand, since jaw deviation is a disharmony involving the maxilla and mandible that is caused by a combination of various factors from hard to soft tissues, a uniform evaluation of these is likely to fail to identify a clear characteristic. Therefore, it is necessary to classify the maxilla, mandible, and soft tissue as separate regions and integrate their results for quantification of jaw deviation and pattern of deviation aspect. Osawa quantified jaw deviation and classified patterns of deviation in the mandible for skeletal mandibular prognathism [21], which is the most frequent and most likely cause of jaw deviation among jaw deformities in Japan [22].

In this study, we aimed to reveal its characteristics by focusing on the yawing and rolling deviation of the maxillary bone, quantifying the aspect of deviation and classifying them based on similarities in three-dimensional maxillary bone morphology using cluster analysis, in skeletal mandibular prognathism.

## Material and method

### Subjects

Subjects consisted of 100 patients (35 males, 65 females; mean age 22 years 5 months ± 8 years 3 months) diagnosed with skeletal mandibular prognathism at the Orthodontic clinic, Niigata University Medical and Dental Hospital from 2009 to 2019. The subjects met the following criteria: no congenital anomalies (e.g., cleft lip and palate), syndromes that affected craniofacial morphology, or no history of traumatic injury. 3D-CT images were taken for all patients during the clinical examination. CT imaging was performed using a Multi-Detector-row CT (Aquilion, Toshiba, Tokyo, Aquilion ONE, Toshiba, Tokyo, Ingenuity CT, Philips, Netherlands), which is available at Niigata University Medical and Dental Hospital, with the patient in the supine position with the mouth closed and positioned parallel to the Reid's Base, at tube voltage of 120 kV and tube current of 54–150 mA. The scanning range was from supraorbital margin to submandibular (including Me), slice thickness was 0.5 mm and 1.0 mm, and slice interval was 0.3 mm and 0.5 mm. After the CT images were converted to Digital Imaging and Communications in Medicine (DICOM) format, they were imported into a personal computer, and MPR images were created and morphometry was performed using the 3D morphometry software ZioCube (Ziosoft, Tokyo).

This study was approved by the Niigata University Ethics Review Committee for Research Involving Human Subjects (Approval No.: 2017–0335).

### Measurements

**Fig.1:**
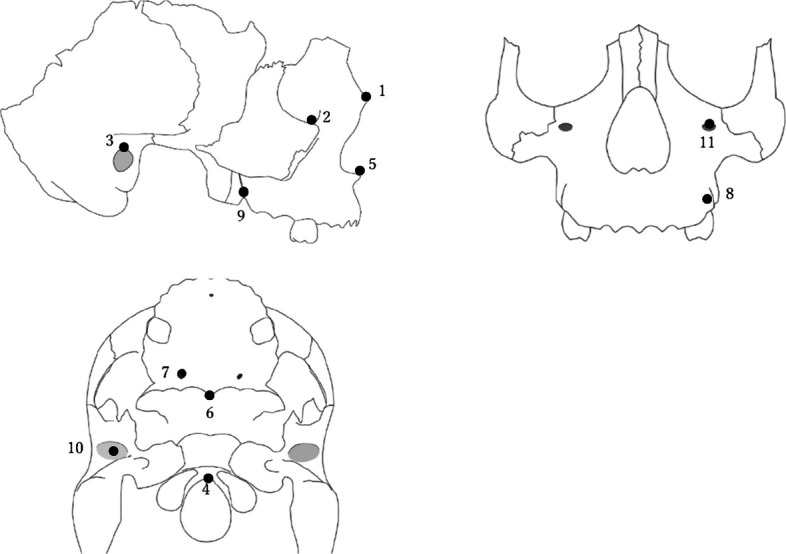
Measurement points. 1) N: Most anterior point of the junction of the nasal and frontal bones; 2) Or: Most inferior point of the inferior orbital rim; 3) Po: Most superior point of external auditory canal; 4) Ba: Most forward point of the large rear head hole; 5) ANS: Most anterior point of the anterior nasal spine; 6)PNS: Most posterior point of the posterior nasal spine; 7)GPF: Center of the opening of the greater palatine foramen; 8)U6: Most outer point of the alveolar bone of buccal cervical area of maxillary first molar; 9)Ptm: Most inferior point of the pterygomaxillary fissure; 10) MF: Most concave point of the mandibular fossa; 11)IoF: Center of the opening of the infraorbital foramen

The setting of the coordinate system for morphometric measurements was performed on the ZioCube morphometric software. For establishing the XYZ Cartesian coordinate system, based on the MPR images of each case created, the horizontal reference plane (XY plane) was first defined as the plane passing through bilateral Orbitales (Or, Or’) and the midpoint of bilateral Porions (Po, Po’). The mid-sagittal reference plane (YZ plane) was defined as the plane perpendicular to the XY plane and passing through the most inferior point on the anterior margin of the foramen magnum occipitalis (Ba) and the most anterior point on the frontal nasal suture (N). Then, the coronal reference plane (XZ plane) was set to be a plane perpendicular to both the XY and YZ planes and passing through Ba. After setting the coordinate system, the following eleven measurement points were set according to the definition of each measurement point shown in Fig. 1 following the method in previous study [23]*.*

- N: Most anterior point of the junction of the nasal and frontal bones.
- Or, Or’: Most inferior point of the inferior orbital rim.
- Po, Po’: Most superior point of external auditory canal.
- Ba: Most forward point of the large rear head hole.
- ANS: Most anterior point of the anterior nasal spine.
- PNS: Most posterior point of the posterior nasal spine.
- GPF, GPF’: Center of the opening of the greater palatine foramen.
- U6, U6’: Most outer point of the alveolar bone of buccal cervical area of maxillary first molar.
- Ptm, Ptm’: Most inferior point of the pterygomaxillary fissure.
- MF, MF’: Most concave point of the mandibular fossa.
- IoF, IoF’: Center of the opening of the infraorbital foramen.

The three-dimensional XYZ coordinate values of each measurement points were mathematically transformed into Affin transforms to determine the three-dimensional coordinates in the XYZ reference coordinate system, and the following measurement items were defined based on the set measurement points.

a ANS-MSP: Distance from the mid-sagittal reference plane to the ANS.
b PNS-MSP: Distance of PNS from the mid-sagittal reference plane.
c (c'). GPF-CP: Distance of GPF (GPF') from the coronal reference plane.
d (d'). U6-CP: Distance from the coronal reference plane to U6 (U6').
e (e'). Ptm-CP: Distance from the coronal reference plane to Ptm (Ptm').
f (f'). MF-CP: Distance from coronal reference plane to MF(MF').
g (g'). IoF-CP: Distance from coronal reference plane to IoF (IoF').
h (h'). GPF-FH: Distance from the horizontal reference plane to GPF(GPF').
i (i'). U6-FH: Distance from the horizontal reference plane to U6 (U6').
j (j'). Ptm-FH: Distance from the horizontal reference plane to Ptm (Ptm').
k (k'). MF-FH: Distance from the horizontal reference plane to MF(MF').
l (l'). IoF-FH: Distance from the horizontal reference plane to IoF (IoF').

Additionally, to grasp the degree of asymmetry, twelve analysis items were set up as shown below, which were added by calculating some of the above measurement items and the absolute values of the left–right difference.

1. ANS-MSP:a
2. PNS-MSP:b
3. GPF-CP-diff:|c–c’|
4. U6-CP-diff:|d-d’|
5. Ptm-CP-diff:|e-e’|
6. MF-CP-diff:|f-f’|
7. IoF-CP-diff:|g-g’|
8. GPF-FH-diff:|h–h’|
9. U6-FH-diff:|i-i’|
10. Ptm-FH-diff:|j-j’|
11. MF-FH-diff:|k-k’|
12. IoF-FH-diff:|l-l’|

Deviation in the “Yawing” direction was evaluated using ANS-MSP, PNS-MSP, GPF-CP-diff, U6-CP-diff, Ptm-CP-diff, MF-CP-diff, and IoF-CP-diff. Deviation in the “Rolling” direction was evaluated using GPF-FH-diff, U6-FH-diff, Ptm-FH-diff, MF-FH-diff, and IoF-FH-diff.

Only one author (C.A.) set the coordinate system and performed all measurements. This analysis method was tested for measurement error before collecting research data. Specifically, 3D CT images from 10 cases measured by the same individual were randomly selected. After an interval of at least one week, the coordinate system was set again and measurements were repeated. Measurement error was calculated using the Dahlberg formula, yielding extremely low values ranging from 0.16 to 0.39.

### Statistical analysis

A cluster analysis using the Ward method was performed using the statistical analysis software R (Ver. 3.3.2, R Foundation for Statistical Computing, Vienna) based on the measured items of deviation in the Yawing direction and in the Rolling direction obtained from each case and created dendrograms. The dendrogram was then used to group the patients. In order to clarify the characteristics of each group, the Steel–Dwass test was used to statistically analyze the means of the analyzed items in each group, with a significance level of *p* < 0.05. Based on the results of the cluster analysis of asymmetry in the Yawing direction and asymmetry in the Rolling direction of the maxilla, each case was reclassified into a group with both characteristics of asymmetry in the Yawing direction and asymmetry in the Rolling direction of the maxilla. Steel–Dwass test was also performed for the means of the analyzed items in each group to clarify the characteristics for each of the reclassified groups, and the results were statistically analyzed.

## Result

### Quantitative classification by cluster analysis

**Fig. 2:**
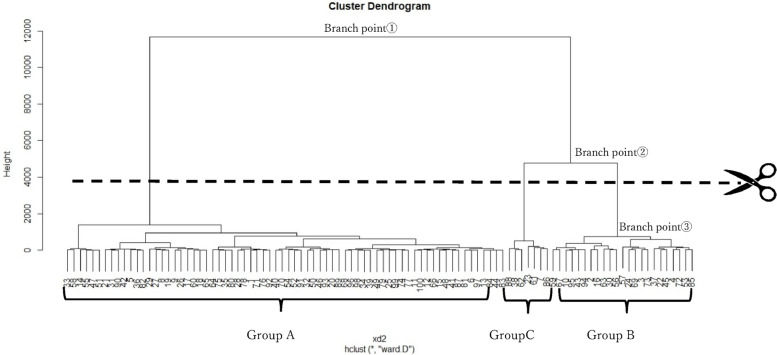
Dendrogram from cluster analysis based on deviation in Yawing direction. Branch point ①: Squared Euclidean Distance 12,000**.** Branch point ②: Squared Euclidean Distance 4500. Branch point ③: Squared Euclidean Distance 1000

The dendrogram obtained by cluster analysis based on seven items (ANS-MSP, PNS-MSP, GPF-CP-diff, U6-CP-diff, Ptm-CP-diff, MF-CP-diff, and IoF-CP-diff) related to the Yawing direction deviation is shown in Fig. 2. Taking into account the number of groups obtained, the Euclidean square distance between the branchpoints of each group, the bias in the number of samples in each group, and the similarity between the groups were taken into account before cutting between Branchpoints ②-③ in the dendrogram, resulting in three groups, A, B and C. The cases classified into each group were 70 patients in Group A, 23 patients in Group B, and 7 patients in Group C.

**Fig.3:**
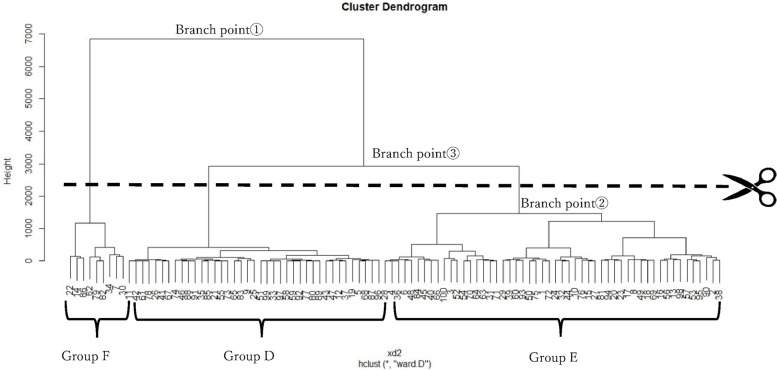
Dendrogram from cluster analysis based on deviation in Rolling direction. Branch point ①: Squared Euclidean Distance 7000 Branch point ②: Squared Euclidean Distance 3000 Branch point ③: Squared Euclidean Distance 1500

The dendrogram obtained by cluster analysis based on the five items related to the deviation in the Rolling direction (GPF-FH-diff, U6-FH-diff, Ptm-FH-diff, MF-FH-diff, and IoF-FH-diff) is shown in Fig. 3. As the same as creating group A to C, the number of groups obtained, the Euclidean square distance between the branchpoints of each group, the bias in the number of samples in each group, and the similarity between the groups were taken into account before cutting between Branchpoints ②-③ in the dendrogram, resulting in three groups, D, E and F. The cases classified into each group were 39 patients in Group D, 52 patients in Group E, and 9 patients in Group F.

### Means, standard deviations for the analyzed items in each group, and between-group comparisons

Table 1 shows the results of the multi-group comparison among the three groups resulting from the cluster analysis for the seven items (ANS-MSP, PNS-MSP, GPF-CP-diff, U6-CP-diff, Ptm-CP-diff, MF-CP-diff, and IoF-CP-diff) related to the deviation in the Yawing direction. The differences between groups in each measured item were as follows.

- ① ANS-MSP: Group A (0.95 ± 0.67 mm) showed significantly smaller values than Group C (1.77± 0.75 mm). Group B (1.31 ± 0.90 mm) was not significantly different from either Group A (0.95 ± 0.67 mm) or Group C (1.77 ± 0.75 mm).
- ② PNS-MSP: Group A (0.66±0.42 mm) showed significantly smaller values than Group B (1.01±0.60 mm). Group C (1.16±0.64 mm) showed no significant difference between Group A (0.66±0.42 mm) and Group B (1.01±0.60 mm).
- ③ GPF-CP-diff: Group A (0.74±0.53 mm) showed significantly smaller values than Group B (1.46±0.72 mm) and Group C (2.33±1.04 mm). No significant difference was observed between Group B (1.46±0.72 mm) and Group C (2.33±1.04 mm).
- ④ U6-CP-diff: Group A (1.22±0.85 mm) showed significantly smaller values than Group B (2.52±1.45 mm) and Group C (3.86±1.46 mm). No significant difference was observed between Group B (2.52±1.45 mm) and Group C (3.86±1.46 mm).
- ⑤ Ptm-CP-diff: Group A (1.10±0.75 mm) showed significantly smaller values than Group B (2.17±0.76 mm) and Group C (3.20±1.23 mm). No significant difference was observed between Group B (2.17±0.76 mm) and Group C (3.20±1.23 mm).
- ⑥ MF-CP-diff: Group A (2.06±1.56 mm) showed significantly smaller values than Group B (4.01±2.54 mm) and Group C (6.04±2.98 mm). No significant difference was observed between Group B (4.01±2.54 mm) and Group C (6.04±2.98 mm).
- ⑦ IoF-CP-diff: Group A (1.19±0.83 mm) showed significantly smaller values than Group B (1.74±1.00 mm) and Group C (4.66±0.97 mm), and Group B (1.74±1.00 mm) also showed significantly smaller values than Group C (4.66±0.97 mm).

**Table 1:** Comparison of analysis items between three groups based on deviation in Yawing direction

							Group A	Group A	Group B
	Group A		Group B		Group C		vs	vs	vs
Analysis item	Mean	SD	Mean	SD	Mean	SD	Group B	Group C	Group C
ANS-MSP	0.95	0.67	1.31	0.90	1.77	0.75	n.s	*	n.s
PNS-MSP	0.65	0.42	1.01	0.60	1.16	0.64	*	n.s	n.s
GPF-CP-diff	0.74	0.53	1.46	0.72	2.33	1.04	***	***	n.s
U6-CP-diff	1.22	0.85	2.52	1.45	3.86	1.46	***	***	n.s
Ptm-CP-diff	1.10	0.75	2.17	0.76	3.20	1.23	***	**	n.s
MF-CP-diff	2.06	1.56	4.01	2.54	6.04	2.98	**	**	n.s
IoF-CP-diff	1.19	0.83	1.74	1.00	4.66	0.97	*	***	**

^*^p < 0.05, **: *p* < 0.01, ***: *p* < 0.001、n.s.: not significant

Group C had the largest value for all analysis items, followed by Group B, and Group A had the smallest value.

On the other hand, the results of multigroup comparisons among the three groups obtained from cluster analysis for the five items related to deviation in the Rolling direction (GPF-FH-diff, U6-FH-diff, Ptm-FH-diff, MF-FH-diff, and IoF-FH-diff) are shown in Table 2. The differences between groups in each measured item were as follows.

- ⑧ GPF-FH-diff: Group D (0.39±0.26 mm) showed significantly smaller values than Group F (1.01±0.94 mm). Group E (0.93±0.59 mm) was not significantly different from Group D (0.39±0.26 mm) or Group F (1.01±0.94 mm).
- ⑨ U6-FH-diff: Group D (0.88±0.45 mm) showed significantly smaller values than Group F (2.58±1.36 mm). Group E (1.24±1.10 mm) was not significantly different from Group D (0.88±0.45 mm) and Group F (2.58±1.36 mm).
- ⑩ Ptm-FH-diff : Group F (2.25±1.98 mm) showed significantly larger values than Group D (1.22±0.80 mm) and Group E (1.98±1.34 mm). Group D (1.22±0.80 mm) and Group E (1.98±1.34 mm) showed no significant difference.
- ⑪ MF-FH-diff: No significant difference was found between Group D (1.21±0.65 mm), Group E (1.70±1.44 mm) and Group F (3.13±2.53 mm).
- ⑫ IoF-FH-diff : Group F (2.23±1.13 mm) showed significantly larger values than Group D (0.65±0.44 mm) and Group E (0.95±0.64 mm). No significant difference was found between Group D (0.65 ± 0.44 mm) and Group E (0.95 ± 0.64 mm).

**Table 2:** Comparison of analysis items between three groups based on deviation in Rolling direction

							Group D	Group D	Group E
	Group D		Group E		Group F		vs	vs	vs
Analysis item	Mean	SD	Mean	SD	Mean	SD	Group E	Group F	Group F
GPF-FH-diff	0.39	0.26	0.93	0.59	1.04	0.94	n.s	***	n.s
U6-FH-diff	0.88	0.448	1.24	1.10	2.58	1.36	n.s	**	n.s
Ptm-FH-diff	1.22	0.80	1.98	1.34	2.25	1.98	n.s	*	*
MF-FH-diff	1.21	0.65	1.70	1.44	3.13	2.53	n.s	n.s	n.s
IoF-FH-diff	0.65	0.44	0.95	0.64	2.23	1.13	n.s	**	*

^*^: *p* < 0.05, **: *p* < 0.01, ***: *p* < 0.001, n.s.: not significant

Group F had the largest value for all analysis items, followed by Group E, and Group D had the smallest value.

### Quantitative classification obtained by both Yawing and Rolling direction deviation features

Subjects were classified into mild deviation, moderate deviation, and severe deviation according to Yawing deviation and Rolling deviation, respectively. In addition, each case was reclassified to a group that had both characteristics of Yawing and Rolling deviation. As a result, the subjects were classified into nine groups, Group 1 through Group 9, as shown in Table 3.

**Table 3:** Results of reclassification based on deviation in Yawing direction and deviation in Rolling direction

		Deviation in Rolling direction		
		**Group D** **(39 subjects)**	**Group E** **(52 subjects)**	**Group F** **(9 subjects)**
Deviation in Yawing direction	**Group A** **(70 subjects)**	Group 1 (31 subjects)	Group 2 (35 subjects)	Group 3 (4 subjects)
	**Group B** **(23 subjects)**	Group 4 (6 subjects)	Group 5 (15 subjects)	Group 6 (2 subjects)
	**Group C** **(7 subjects)**	Group 7 (2 subjects)	Group 8 (2 subjects)	Group 9 (3 subjects)

As a breakdown of each group, Group 1, which showed no deviation in both Yawing and Rolling direction, i.e., no asymmetry, had 31 patients. In contrast, in the group of cases with deviation in either or both Yawing and Rolling direction, i.e., asymmetry, there were 69 patients (Groups 2 to 9). The most frequent group with asymmetry was Group 2 with 35 patients, followed by Group 5 with 15 patients, Group 4 with 6 patients, Group 3 with 4 patients, and Group 9 with 3 patients. The fewest frequent groups with asymmetry were Group 6, Group 7, and Group 8, which belonged to only two patients each. The means and standard deviations of the analyzed items in each group are shown in Table 4, and the results of the multigroup comparisons are shown in Table 5.

- ① ANS-MSP: No significant difference was found among all groups.
- ② PNS-MSP: No significant difference was found among all groups.
- ③ GPF-CP-diff: Group 4 (1.60 ± 0.64) was significantly higher than Group 1 (1.23 ± 0.68) and Group 2 (1.17 ± 0.66).
- ④ U6-CP-diff: Group 4 (1.60 ± 0.64) was significantly higher than Group 1 (1.23 ± 0.68) and Group 2 (1.17 ± 0.66).
- ⑤ Ptm-CP-diff: No significant difference was found among all groups.
- ⑥ MF-CP-diff: Group 5 (3.57 ± 2.85) was significantly higher than Group 1 (2.02 ± 1.44) and Group 2 (2.07 ± 1.55).
- ⑦ IoF-CP-diff: No significant difference was found among all groups.
- ⑧ GPF-FH-diff: Group 2 (0.93 ± 0.56) was significantly higher than Group 1 (0.40 ± 0.27).
- ⑨ U6-FH-diff: No significant difference was found among all groups.
- ⑩ Ptm-FH-diff: No significant difference was found among all groups.
- ⑪ MF-FH-diff: No significant difference was found among all groups.
- ⑫ IoF-FH-diff: No significant difference was found among all groups.

**Table 4:** Mean values and standard deviation in each group

	Group 1		Group 2		Group 3		Group 4		Group 5		Group 6		Group 7		Group 8		Group 9	
Analysis item	Mean	SD	Mean	SD	Mean	SD	Mean	SD	Mean	SD	Mean	SD	Mean	SD	Mean	SD	Mean	SD
ANS-MSP	1.04	0.78	0.83	0.57	1.25	0.39	1.41	0.74	1.13	0.94	2.02	0.28	1.81	0.08	2.15	0.62	1.48	0.93
PNS-MSP	0.60	0.35	0.69	0.49	0.60	0.24	0.79	0.48	1.01	0.62	1.49	0.26	1.40	0.65	1.35	0.66	0.88	0.50
GPF-CP-diff	0.60	0.44	0.80	0.58	1.01	0.38	1.78	0.87	1.39	0.57	1.14	0.78	3.04	0.99	1.24	0.39	2.59	0.77
U6-CP-diff	1.23	0.68	1.17	0.66	1.15	0.55	1.60	0.64	3.02	0.92	2.25	0.45	2.92	1.91	3.28	0.90	3.38	0.68
Ptm-CP-diff	0.99	0.80	1.11	0.88	1.09	0.54	2.44	0.72	2.17	1.42	1.70	1.25	2.85	1.04	4.02	0.21	4.37	1.83
MF-CP-diff	2.02	1.44	2.07	1.55	1.25	0.88	5.28	1.34	3.57	2.85	4.62	0.83	3.01	0.53	6.97	3.06	7.43	2.37
IoF-CP-diff	1.15	0.75	1.17	0.79	1.00	0.24	2.20	1.28	1.61	1.00	2.41	0.37	4.14	0.11	5.30	1.45	4.58	0.57
GPF-FH-diff	0.40	0.27	0.93	0.56	0.70	0.66	0.32	0.23	0.91	0.69	1.53	1.44	0.96	0.08	0.32	0.25	1.15	0.62
U6-FH-diff	0.83	0.80	2.11	1.38	1.70	2.07	0.98	0.73	1.28	1.21	1.12	0.65	0.21	0.40	2.00	0.49	3.00	2.08
Ptm-FH-diff	1.19	0.49	1.28	1.09	2.26	1.27	1.10	0.39	1.88	1.12	3.87	0.85	0.53	0.14	1.28	0.09	2.91	0.81
MF-FH-diff	1.17	0.67	1.43	1.35	3.61	3.11	1.48	0.55	2.29	1.40	2.86	1.65	1.99	1.85	0.93	0.31	2.67	1.99
IoF-FH-diff	0.64	0.47	0.93	0.68	2.07	1.27	0.67	0.26	0.90	0.49	1.87	1.27	1.54	0.45	0.60	0.59	2.68	0.51

**Table 5:** Results of each group’s Steel–Dwass test for only those that recognized correlation

Analysis item	Subject	P Value	Significance
GPF-CP-diff	Group 1 vs Group 4	0.0016	*
	Group 2 vs Group 4	0.0348	**
U6-CP-diff	Group 1 vs Group 4	0.0031	**
	Group 2 vs Group 4	< 0.0001	***
MF-CP-diff	Group 1 vs Group 5	0.0095	**
	Group 2 vs Group 5	0.0090	**
GPF-FH-diff	Group 1 vs Group 2	0.0006	***

^*^: *p* < 0.05, **: *p* < 0.01, ***: *p* < 0.001, n.s.: not significant

**Fig. 4:**
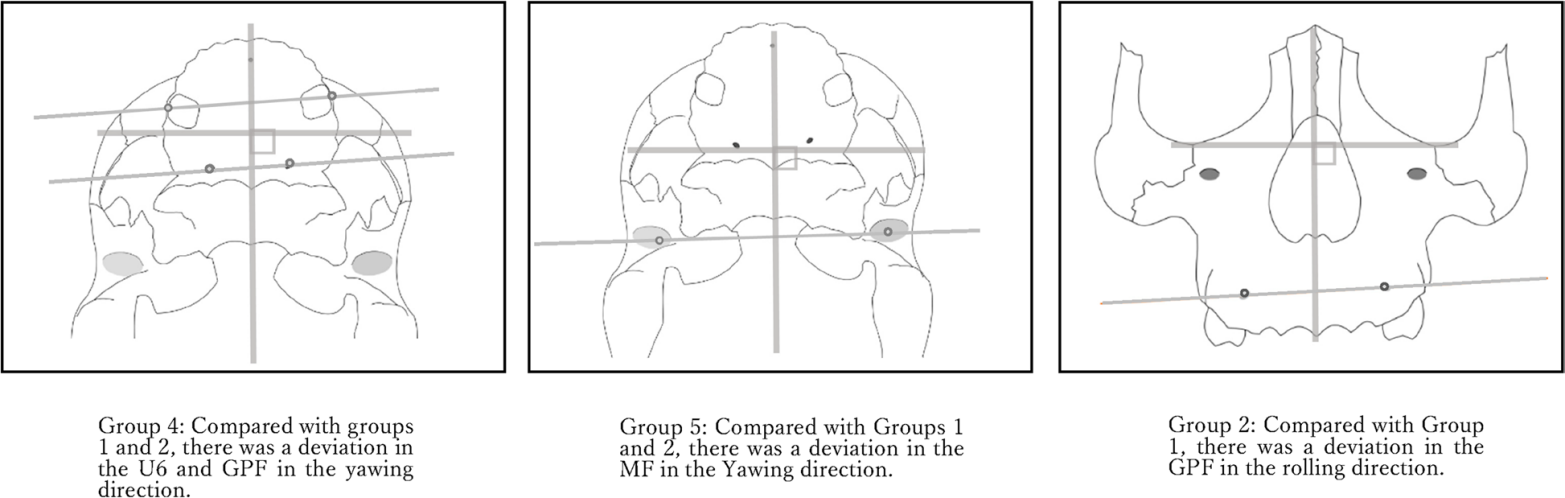
Conceptual diagram representing the characteristics of the groups in which significant differences occurred

According to the results of the above statistical analysis, the characteristics of the nine groups classified in this study can be summarized as follows. For the groups that showed significant differences, a conceptual diagram shown in Fig. 4 was created based on the characteristics.

- Group 1 (31 patients): The maxillary morphology was nearly symmetrical in both Yawing and Rolling directions.
- Group 2 (35 patients): In the Yawing direction, the maxillary morphology is almost symmetrical. In the Rolling direction, there is a deviation especially at the GPF.
- Group 3 (4 patients): In the Yawing direction, the maxillary morphology is nearly symmetrical. In the Rolling direction, moderate to severe deviation.
- Group 4 (6 subjects): In the Yawing direction, the maxillary morphology is deviated especially at U6 and GPF. In the Rolling direction, they are nearly symmetrical.
- Group 5 (15 patients): The maxillary morphology is deviated in the Yawing direction, especially at the MF. In the rolling direction, there is a slight deviation.
- Group 6 (2 patients): In the Yawing direction, the maxillary morphology is mildly deviated. In the Rolling direction, there is moderate to severe deviation.
- Group 7 (2 patients): In the Yawing direction, the maxillary morphology is moderately to severely deviated. In the Rolling direction, nearly symmetrical.
- Group 8 (2 subjects): In the Yawing direction, the maxillary morphology is moderately to severely deviated. In the Rolling direction, the deviation is mild.
- Group 9 (3 patients): Both in the Yawing and Rolling directions, the maxillary morphology is moderately to severely deviated.

## Discussion

### Subjects and methods

There have been various studies on the origins of jaw deviation [8, 24–27], however, it is not yet sufficiently understood because of the many variations in its morphology. In this study, we focused on the Yawing and Rolling deviation of the maxillary morphology and attempted to quantify and classify the jaw deviation by organizing the aspects of jaw deviation using cluster analysis. To classify the data, the cluster analysis was used, which is considered to be suitable for the classification of complex jaw deviations and capable of classifying populations based on objective numerical criteria from a variety of data[20]. There have been several reports using cluster analysis to classify the characteristics of maxillofacial morphology. Hwang et al. [26] found that 100 patients diagnosed with facial asymmetry using frontal cephalograms could be classified into five groups that showed clear characteristics by cluster analysis. On the other hand, Baek et al*.* [24] reported that 43 patients diagnosed with facial asymmetry using three-dimensional computed tomography (3D-CT) images could be classified into four groups by cluster analysis. However, one of the four groups obtained in the results of Baek et al. consisted of two persons, suggesting that the group may not have been clearly classified statistically. In this study, patients with skeletal mandibular prognathism with anterior-posterior discordance of the upper and lower jaw without congenital abnormalities such as cleft palate were included. This is because skeletal mandibular prognathism is the most frequent form of jaw deformity in Asian population including Japanese, and because it is considered to be the most likely to cause jaw deviation [22], and therefore the aspect of deviation was considered to be easy to grasp. Based on the report of Baek et al. the number of patients in this study was set at 100 in order to obtain a more detailed classification and a clinically useful classification for jaw deviation in the maxilla.

The setting of the midsagittal reference plane is extremely important to properly evaluate the three-dimensional aspect of jaw deviation. Therefore, it is essential to select an anatomical reference point that is less susceptible to jaw deviation [15]. The setting of the median sagittal reference plane should be a clinically acceptable and reproducible reference plane, according to Thiesen et al. [28]. After considering reference points that are less affected by jaw deviation and are easy to set and reproducible, the midsagittal reference plane in this study was defined as a plane that is perpendicular to the horizontal reference plane (FH) and passes through N and Ba. N and Ba were considered to be less susceptible to jaw deviation because of their proximity to the cranial base and their medial location and distance from the measurement point. It is also possible that the FH plane may be affected by deviation in the temporal bone [29]. However, the FH plane was selected as one of the reference planes in this study because images with the FH plane parallel to the floor are often used when evaluating facial features and are frequently used clinically, especially by orthodontists. A report using the ANS as a reference point for setting the midsagittal reference plane is recognized previously [30]. However, since the purpose of this study is to evaluate the deviation of the maxilla itself, we decided to use the measurement points of a stable structure close to the cranium for setting each reference plane instead of using the reference points of the maxilla. Traditionally, maxillary deviation has been evaluated mainly by focusing on the horizontal occlusal plane canting, and there are many references in the literature that use the cusp or the tooth axis of the maxillary first molar as the reference point or line [31]. However, in this study, in order to evaluate the aspect of deviation of the maxillary bone itself, the reference points were set to the anatomical points of the maxilla, palatine bone, and temporal bone. Vertical deviation of the maxilla is sometimes evaluated by J-J' in the frontal cephalogram [7], but J point in the frontal cephalogram is a point on the graphic drawing and is difficult to define three-dimensionally, so we excluded it from our evaluation. We attempted to evaluate the morphology of the maxilla using ANS, PNS, GPF, Ptm, U6, MF, and IoF, which are considered more susceptible to influence by the symmetry and positional deviation of the maxilla itself, based on previous literature that evaluated the morphological deviation of the maxilla [24, 31]. The mandibular fossa, one of the measurement points that we employed, is a landmark on the temporal bone adjacent to the zygomatic bone (part of the nasomaxillary complex) via the zygomatic suture. In cases of mandibular deviation, extensive displacement is often observed. Since establishing three-dimensional landmarks on the zygomatic bone proved difficult due to its anatomical features reflecting maxillary deviation, we decided to use the mandibular fossa of the adjacent temporal bone as a useful three-dimensional landmark for analysis. Furthermore, the mandibular fossa is the only point connecting the maxilla and the mandible. Osawa et al. [21] have classified the patterns of mandibular displacement, and this landmark is considered to be of great importance for future research into the relationship between mandibular and maxillary displacement patterns.

In this study, only distance measurements were used as measurement items. The reason for this is that if distance and angle measurements were mixed in the measurement items, the weighting would be different when performing cluster analysis, and in addition, the characteristics of the obtained classification would become more complex, making it more difficult to recognize the deviation pattern. Furthermore, to determine asymmetry, the absolute difference between left and right distance measurements was used in this study. The direction of deviation was not included in this study. This is because preliminary research conducted prior to the study indicated that if the direction of deviation was taken into account in the measurement, the clustering would be further subdivided according to the direction of deviation, making it difficult to understand the characteristics of each group. Therefore, the results of this study using left–right differences in measurements are considered to reflect the degree of jaw deviation, regardless of the direction of the deviation. However, the characteristics of cases with the direction of deviation, such as cases of inverse cant, have not been investigated, and further studies will be necessary.

### Cluster analysis

The dendrogram obtained by cluster analysis is bifurcated based on Euclidean square distance. The closer the distance between the bifurcation points in the dendrogram, the better the approximation between arbitrary clusters, and the farther the distance between the bifurcation points, the lower the similarity. This means that the shorter the length of the vertical axis of the dendrogram, the higher the similarity between clusters. Therefore, when dividing the target into groups, it is necessary to determine the cut-off sites of the dendrogram so that the Euclidean square distance is long enough and the number of clusters is large enough to have each characteristic in each cluster. In this study, the dendrogram obtained by cluster analysis of the Yawing deviation of the maxilla was first divided into two clusters at a Euclidean square distance of about 12,000 (Fig. 2 ①). One of the clusters was further divided into two clusters at a Euclidean square distance of about 4500 (Fig. 2 ②). On the other hand, the dendrogram obtained by cluster analysis of the Rolling deviation of the maxilla was first divided into two clusters at a Euclidean square distance of about 7,000 (Fig. 3 ①). One of the clusters was further divided into two clusters at a Euclidean square distance of about 3,000 (Fig. 3 ②) In both Figs. 2 and 3, if the dendrogram is cut below branch ③, the Euclidean square distance becomes shorter. This leads to a high similarity of each cluster, which obscures the morphological characteristics. Therefore, the number of clusters was determined to be three each when the dendrogram was cut between branches (2) and (3) in Fig. 2 and Fig. 3, respectively, which is suitable for clinical application because it appropriately reflects the characteristics of the anatomical structure without becoming overly complex for diagnosis of orthognathic cases.

### Classification of jaw deviation

This study was focusing on the maxilla, whose deviation is considered to affect the treatment plan of surgical orthodontic treatment [32], in order to quantify the aspect of jaw deviation and group the patients according to the characteristics of deviation. Since jaw deformities with facial asymmetry often involve the upper and lower jaws, both jaws should normally be considered and evaluated together. However, as the number of factors required for cluster analysis increases, the number of required cases also increases significantly. In other words, if the classification is based on a small number of cases, it may yield only a group with no clear morphological features. Therefore, we focused on only the maxilla of skeletal mandibular prognathism, and an attempt was made to classify it from two points of view: deviation in the Yawing direction and deviation in the Rolling direction, which are considered to be directly related to malocclusion disharmony in cases of facial asymmetry.

Traditionally, maxillary deviation has been evaluated using the occlusal plane inclination at the maxillary first molar and the J-J' angle on the frontal cephalogram. However, the cant of occlusal plane at the maxillary first molar is influenced by bucco-lingual tooth inclination, and the J-J' angle on the cephalometric profile is a two-dimensional point measurement. Therefore, both are considered unreliable for evaluating maxillary cant in three-dimensional analysis. So, to evaluate the displacement of the maxilla itself, we decided to use three-dimensional landmarks to assess displacement in the yawing and rolling directions. Regarding rolling displacement, since we changed the type of landmarks used compared to conventional evaluation methods, we believe this allows for assessment from a broader perspective than the conventional concept of canting.

In this study, based on the results of cluster analysis of Yawing and Rolling deviation of maxillary morphology, individual cases were reclassified to a group with both characteristics with a characteristic morphology in three dimensions. As results showed, Group 1 became "the group whose maxillary morphology is almost symmetrical in both Yawing and Rolling directions and shows no deviation," while Groups 2 through 9 became "the group whose maxillary morphology shows deviation in either or both Yawing and Rolling directions. The number of subjects categorized in Group 1, which did not show any deviation of the maxilla, was 31 cases, accounting for about 30% of the total number of subjects. In contrast, Groups 2 through 9, which included approximately 70% of the remaining subjects, were indicated as cases with some form of asymmetry or deviation. In a report by Severt et al. [33] on two-dimensional morphometry in patients with dental and facial deformities, clinically evident facial asymmetry was found in 34% of all patients, and vertical asymmetry in the occlusal plane was found in 41%. The percentage of maxillary deviation in the Rolling direction in the present study was 61%, which is higher in comparison. This may be due to the fact that the three-dimensional evaluation identified cases with not only horizontal occlusal plane inclination but also deviations in the greater palatine foramen, pterygopalatine fossa, and infraorbital foramen, which have not been the focus of attention when discussing the relationship between jaw deviation and the maxilla. In other words, evaluating the aspect of deviation in the maxilla reveals the asymmetry of these anatomical structures. This suggested that they could be used as landmarks to define the jaw deviation of the maxilla. Furthermore, since these measurement points are also areas that can be improved by orthognathic surgery, they could be used to diagnosis and evaluate treatment results.

Eight groups (Group 2 to Group 9) with deviation of maxillary morphology in both or either Yawing or Rolling direction could be classified. Group 2 had a nearly symmetrical maxillary morphology in the Yawing direction. However, in the Rolling direction, there were differences in the vertical position of the foramen magnum, especially between the right and left sides. This may be similar to cases in which J-J' inclination has been conventionally recognized, but it is not a matter of speculation because the points of measurement are different. However, cases in which a left–right cant of the palatal region is present are occasionally observed clinically. Considering that the percentage of subjects classified in Group 2 was the highest among all groups at 35%, it was inferred that this is a relatively frequent aspect of maxillary deviation. Group 3 was classified as a separate group because of its greater deviation in the Rolling direction compared to Group 2. However, the frequency of Group 3 was low at 4% of the total and was considered a relatively rare deviation pattern. Because the number of cases in this group was only four, there were no significant differences among the other groups. However, when comparing the mean values with the other groups, MF-FH had the largest deviation, and Ptm-FH and IoF-FH also had the second largest deviation, possibly reflecting the deviation of the upper part of the maxilla above the alveolar region. Group 4 was characterized by no deviation in the Rolling direction, but only in the Yawing direction, with particularly large left–right differences in the anteroposterior position of the U6 and GPF, that is, the posterior maxillary region. No significant difference was observed in MF, Ptm, and IoF, and significant deviation was observed in U6 and GPF, suggesting that Group 4 is mainly deviated in the Yawing direction near the molar region of alveolar process. This means that there is little deviation in the anterior part of the maxilla, but deviation is present as the posterior alveolar process twists. In cases with this feature, it may be difficult to recognize the deviation itself because the deviation is confined to the posterior portion of the dentition. However, the left–right positioning of the posterior region of the upper and lower dental arches should be considered in treatment. Group 5 showed moderate deviation of maxilla in the Yawing direction, especially in the anteroposterior position of the mandibular fossa, and mild to moderate deviation in the Rolling direction. Since the position of the mandibular fossa, the position of the entire mandible relative to the cranium, is itself deviated, the possibility of an anteroposterior deviation in the left and right mandibular rami are also considered. It was suggested that this may be a deviation pattern similar to the "unilateral condylar hyperplasia asymmetry" reported by Baek et al. [24] and the "roll-type asymmetry" reported by Tyan et al. [31]. As it is impossible to improve the position of the mandibular fossa itself by orthognathic surgery, group 5 cases may be suitable for improving facial asymmetry by adding a left–right difference in postoperative mandibular morphology in order to camouflage the anterior–posterior position of the mandibular fossa. Groups 6–9 were both groups that contained only two or three cases, and no features were identified that differed significantly from the other groups. However, each group was considered to have a complex morphology with deviations in both the Yawing and Rolling directions of the maxilla. Both Yawing and Rolling directions were classified into three patterns each in the cluster analysis. For both directions, deviations were classified into mild, moderate, and severe patterns. Clinically, severe cases are rare, and combining the Yawing and Rolling directions resulted in a group with a small number of subjects.

In this study, each of the three groups obtained from the cluster analysis focusing on the Yawing and Rolling deviation of the maxillary morphology were reclassified into nine groups according to their respective characteristics. Furthermore, attempts were made to classify the characteristics of each group through statistical group comparisons. The results showed that some groups could be clearly characterized as statistically significant differences, while others showed no statistical differences among some of the group comparisons. In particular, Groups 3, 6, 7, 8, and 9 showed no significant differences. As for the characteristics of each group, Group 3 showed almost symmetrical maxillary morphology in the Yawing direction and moderate to severe deviation in the Rolling direction, Group 6 showed mild deviation in the Yawing direction and moderate to severe deviation in the Rolling direction, Group 7 showed moderate to severe deviation of maxillary Group 7 showed moderate to severe deviation of maxillary morphology in the Yawing direction and almost symmetrical in the Rolling direction, Group 8 showed moderate to severe deviation of maxillary morphology in the Yawing direction and mild deviation in the Rolling direction, and Group 9 showed moderate to severe deviation of maxillary morphology in both Yawing and Rolling directions. Groups 3, 6, 7, 8, and 9 had fewer than 5 cases in each group, suggesting that the small number of cases likely affected the statistical analysis. In this study, the number of cases was set at 100 to avoid such a small group as much as possible, but as a result, several groups with less than 5% of the total number of cases were found. Thus, the possibility cannot be denied that the number of subjects was insufficient to be classified into 9 groups. However, there were three groups (groups 2, 4, and 5) with clearly characterized patterns for the maxillary deviation pattern. The other five groups (Groups 3, 6, 7, 8, and 9) had relatively few cases. They were found to be severely deviated cases, either in the yawing or rolling direction, or a combination of both. The results showed that 30% of the cases had no clear deviation of the maxilla, 50% of the cases had a tendency of deviation, and 20% of the cases had a complex deviation pattern that was not uniform. Furthermore, the results of our study, in which several groups were formed to which a small number of cases belonged, could reflect the many variations in jaw deviation. It is assumed that further analysis with a larger number of cases will clarify the characteristics of the group of cases that could not be statistically analyzed in this study due to the small number of applicable cases.

Until now, there has been no three-dimensional analysis of maxillary deviation, and the details of the deviation pattern of the maxilla have not been clarified. When the maxillofacial structure shows deviation three-dimensionally, the cause may be both abnormal position of the maxilla and deformation of the maxilla itself, but orthognathic surgery applied in surgical orthodontic treatment mainly improves abnormal position, especially antero-posterior dimension of the maxilla. In this study, quantification of maxillary deviation patterns was attempted from two perspectives: asymmetry in the Yawing direction and asymmetry in the Rolling direction of the maxilla, focusing mainly on positional abnormalities, in consideration of their application to actual treatment. In addition to the previously reported mandibular deviations, this study has made it possible to depict maxillary deviation patterns, which will greatly contribute to the quantification of jaw deviations in the maxillofacial region. Furthermore, the quantification of the maxillary deviation pattern in our study may be useful in the selection of surgical procedures for the maxilla in cases of jaw deformity with deviation, and in examining the morphological changes and stability after treatment. In the future, we intend to retrospectively evaluate and study the postoperative changes and postoperative stability of facial asymmetry cases using this classification in order to provide higher quality surgical orthodontic treatment.

## Conclusion

Quantification and classification of jaw deviation were attempted in 100 patients with skeletal mandibular prognathism by cluster analysis, focusing on two aspects of maxillary morphology: asymmetry in the Yawing direction and asymmetry in the Rolling direction, using three-dimensional CT images in this study. As a result, they were classified into nine groups, four of which could be identified as groups with clearly defined morphological characteristics.

This study found that the anterior–posterior and vertical positions of the foramen magnum, the anterior–posterior position of the mandibular fossa, and the anterior–posterior position of the alveolar bone of the buccal cervical region of the maxillary first molar were useful for the quantitative analysis of maxillary deviation. Patterning the aspect of maxillary deviation could be identified, by using four groups of these morphological characteristics. This suggests the possibility of using them as effective criteria for diagnosis and treatment planning for surgical orthodontic treatment.

## Data Availability

The datasets generated during and/or analyzed during the current study are available from the corresponding author on reasonable request.

## Abbreviations

MPR: Multiplanar reconstruction
FH: Frankfurt horizontal
CT: Computed tomography
2D: Two-dimensional
3D: Three-dimensional
